# Trophic Nerves

**Published:** 1887-08

**Authors:** 


### *TROPHIC NERVES.*

The actual demonstration of the existence of trophic nerve fibres, apart from vaso-motor fibres, has not until recently been made, although many evidences pointing to that conclusion have been put forward. Dr. Joseph, of Berlin, in a number of experiments on cats, produce facts which go to prove the existence of trophic nerve fibres in the peripheral nerves. The complication of vascular changes induced by the removal of the vaso-motor fibres from a part must be excluded in order to prove definitely that any nutritive changes occurring after section of its nerve are due to the loss of trophic influence. These conditions were carefully considered, and in order to avoid any vaso-motor changes, the second cervical nerve was selected for section, which, as will be seen, contains no vaso-motor fibres. This nerve was used in all the experiments. Mere section of the trunk of the nerve produced no effect, as reunion rapidly took place, so that it became necessary to remove a considerable portion of the trunk to prevent this occurrence. In some of the animals the ganglion on the posterior nerve-root was also removed. The changes induced were limited to the area of distribution of the nerve, and in those where the ganglion was removed similar changes occurred in the cutaneous area of distribution of the fifth cranial nerve of the corresponding side, coming on in different animals at a period varying from five to twenty-seven days. The changes consisted in loss of hair, at first localized, afterwards gradually extending, situated above and behind the ear where the trunk was only severed; above the eye and over the cheek, where part of the trunk and the ganglion on the posterior root were removed.

After a time complete baldness and a shiny atrophied state of the skin made their appearance. No increased vascularity, inflammation or any other change whatsoever could be discovered in the skin. The hair was examined for parasites, but none could be found. Microscopic examination of the skin failed to detect anything beyond atrophy of the hair follicles; there was no evidence of inflammatory or other changes. From these experiments, Dr. Joseph concludes that trophic nerve fibres exist apart from vaso-motor fibres, and entirely independent of them; and he explains the similar changes in the area of distribution of the fifth cranial nerve by assuming that it derives its trophic supply from the ganglion on the posterior root of the second cervical nerve from which fibres join the ascending root of the fifth.—*Virchow's Archiv*, Bd. cvii., Hft. 1.—*The Practitioner*, July, 1887.

